# Generation Z Within the Workforce and in the Workplace: A Bibliometric Analysis

**DOI:** 10.3389/fpsyg.2021.736820

**Published:** 2022-02-01

**Authors:** María Dolores Benítez-Márquez, Eva María Sánchez-Teba, Guillermo Bermúdez-González, Emma Sofía Núñez-Rydman

**Affiliations:** ^1^Department of Applied Economics (Statistics and Econometrics), Faculty of Economics and Business, University of Malaga, Málaga, Spain; ^2^Department of Business Management, Faculty of Economics and Business, University of Malaga, Málaga, Spain; ^3^Department of Business Management, Faculty of Commerce and Management, University of Malaga, Málaga, Spain

**Keywords:** Generation Z, workplace, workforce, bibliometric review, SciMAT, thematic cluster

## Abstract

This article aims to improve the knowledge on Generation Z as employees within workforce and in the workplace, as well as on the main thematic trends that drive the research on the topic. To this end, and using bibliometric techniques, a sample of 102 publications on this subject from Web of Science between 2009 and 2020 is analyzed. Research discusses the most published and most cited authors and journals to have a broad view of the context of the subject. Later, through a longitudinal view, the study mainly focuses on analyzing the evolution of thematic clusters, to assess the progress of the themes, as well as the network around the principal motor cluster of each period. The obtained results suggest a hardly developed topic, which started to draw attention in 2018, while still having a wide margin for growth. The core of research on the topic has evolved around “Generation-Z” “generations,” “workplace,” “management” and “attitudes,” “leadership,” “career,” or “learning-teaching-education,” although a low keyword stability among periods was noted. There is a need for further development on a variety of aspects regarding this generation and the labor market, as the study shows a clear orientation toward management and generational diversity within the workplace.

## Introduction

A number of recent studies examine the characteristics of Generation Z (Gen Z) individuals (Gen Zers), their values (Maloni et al., 2019; Cresnar and Nedelko, 2020), their attitudes toward work and organizations (Barhate and Dirani, 2021), the way they adapt to the workplace (Chillakuri, 2020), and even distinguishing intragenerational variants within this cohort (Scholz, 2019; Leslie et al., 2021), as well as its similarities and differences with other generations (Hernaus and Poloski Vokic, 2014; Klopotan et al., 2020; Mahmoud et al., 2021), but mostly with Generation Y (Raslie and Ting, 2021). Given the need to adapt in the workplace not only for the latest generation, but for the cohesion and cooperation between generations, this adds extra difficulty to the human resources management (HRM), and to an efficient workflow and environment in the workplace.

The purpose of this article is to disclose the thematic research trends on the aforementioned topic, through a review of the existing literature on Gen Z as employees within the workforce and in the workplace. This article delivers a pioneering topic to which no research has specifically focused before. The contribution of this research will allow a further understanding and an increased knowledge on how Gen Z is related to the workforce and in the workplace. In addition, the study will create supporting material for future research, as well as helping the HRM to better address the needs of Gen Zers and bring higher value to the organization. Thus, a bibliometric assessment has been elaborated to highlight the number of publications, the most notorious authors, and the most impactful journals. Additionally, quantitative research was elaborated, a longitudinal analysis was developed, as well as a visualization of the data on the most relevant themes of research is disclosed for the different periods considered.

The contextualization of the study is described consequently, including the characteristics of Gen Z and their general expectations of jobs and employers, and the current trends and adaptation practices of HRM and organizations. The third part will be focused on the methods used for the bibliometric analysis, including the search strategy, sample, and software. Thereafter, the results of the analysis are stated on the activity related to the topic, the evolution of the keywords, a thematic longitudinal analysis, and eventually, a period-by-period strategic map analysis. It will discuss the implications, future research suggestions, and limitations of the paper, and finally, conclusions will be described.

## Contextualization of the Study

### Generation Z

Generation Z is the generation born from mid-1990s to early 2010s, where the exact dates vary depending on the chosen author, but most commonly is the 1995–2010. Gen Z is known to be the first true “digital native” generation (Lanier, 2017), as they have been born and have been grown in a digital and technological environment, learning how to use technology, and interacting in social networks since the very young age, and even tend to be seen as addicted to technology. The members of this generation have also been called “Gen Zers,” “post-Millennials,” or “iGen” (Magano et al., 2020).

As its main characteristics, Gen Zers are defined as highly ambitious and self-confident (Pataki-Bittó and Kapusy, 2021). At the same time, they are said to be realistic and accept whatever is given (Scholz, 2019). Gen Z is entrepreneurial (Magano et al., 2020), even more than Generation Y (Lanier, 2017). This generation seems to be motivated by finding their dream job and opportunities to expand their skills (Magano et al., 2020), leading to believe they will switch jobs more frequently than other generations before them, and if they do not like something, they are ready to change immediately (Csiszárik-Kocsír and Garia-Fodor, 2018). Other motivation drivers for this cohort are roots on advancement opportunities, increased salary, a meaningful work, and a good team (PR Newswire, 2014; Csiszárik-Kocsír and Garia-Fodor, 2018).

When looking at how Gen Z is said to think and act, it is highlighted that they are not only more aware and informed about what is going on in the world than previous generations, but they have shown to be financially conscious (Sladek and Grabinger, 2014). Moreover, their consumption is more ethical, and they have “greater freedom of expression and greater openness to understanding different kinds of people” (Francis and Hoefel, 2018, p. 2), having shown to be oriented to others (Magano et al., 2020). This broad view of life gives Gen Z a unique perspective on understanding others, while trying to stay true to themselves, their values, and their goals.

While there is a prototype of this generation with mostly common characteristics and attitudes, among approximately 15 different age groups, it is evident that “one size does not fit all.” Moreover, the earliest works on Gen Z had been conducted almost only in the United States, leading to a biased perception of this generation (Scholz, 2019). There exist supporting studies on intragenerational differences. These revealed how the visions, preferences, and features of Gen Zers vary by regions (Scholz, 2019), or even by their workplace perceptions (Leslie et al., 2021), depending on external situational factors such as events, crises, technology, or trends of their youth.

This generation cohort has been surrounded by a global financial crisis, times of terrorism, political uncertainty, and an almost irreversible climate crisis. At the same time, Gen Z has lived in an increasingly globalized world, with the ease of a same currency around the EU and free mobility through its member states in the case of Europe. All these factors have influenced how Gen Z has forged their personality, their vision, and has made them highly adaptable to the global world (Magano et al., 2020).

It must be taken into account that part of Gen Z is already working, some are currently entering the workforce—more than what is expected because of the pandemic—and others are still on formation. Mainly, Gen Zers have started entering the labor market in the last years. Their introduction to the workforce has been challenging, being disturbed by a pandemic, its economic downturn, and its social and labor implications. There are only a couple of studies that address the impacts of coronavirus disease 2019 (COVID-19) on Gen Z in the labor market (Sakdiyakorn et al., 2021), but the number of articles relating the pandemic and Gen Z is expected to increase when the real effects are known after the return of most of the employees to the workplace and once the pandemic has ended. These downturns have not prevented the cohort of having high expectations about their work (Snieska et al., 2020), as well as having a well-defined career development plans (Barhate and Dirani, 2021).

According to a Deloitte report (O’Boyle et al., 2017, p. 10), Gen Z, with eyes on the workplace, is expected to introduce high technology skills, while some researchers are not completely sure about their interpersonal communication and relationship skills. These researchers also found out that the majority of “Gen Z professionals prefer a multidisciplinary and global focus to their work.” Additionally, it is said that Gen Zers are affected by the belief that companies usually use and care about employees only when they are needed (Scholz, 2019).

Generation Z is said to change jobs more frequently; thus, HR does not only have to worry about how to attract the new generation, but how to focus their efforts on giving Gen Zers what they need to stay in the company. Considering the scant research done in terms of what attracts Gen Zers toward companies, it is said that Gen Z is enticed by the work flexibility and a balance with their life outside the workplace. They seek direct contribution to the company, they desire to have an impact on the outcomes, they are driven by an entrepreneurial mindset, and an already established and known company is a plus (Randstad Canada, 2014).

Furthermore, in terms of employee retention, there are some common aspects to this generation (PR Newswire, 2014; Randstad Canada, 2014): they value honesty over anything else in their leaders, they prefer face-to-face communication with their superiors, they enjoy open dialog, as well as they have a strong desire to be listened to their ideas and to be valued for their opinions by their managers, and additionally, they expect social responsibility. Now the question is, are companies delivering these qualities to their employees? If not, why is it taking so long to adapt to the newest generation? Are firms considering organizational change to address the desires of the Gen Z?

### Generation Z Within the Workforce and the Workplace

Throughout the years, the workforce has been evolving, and has been affected by multiple events, such as the Great Recession and more recently, the COVID-19 pandemic. Similarly, the workplace has developed new dynamics, from separated spaces according to the department, to current open spaces where different departments share resources and knowledge in a faster and more efficient way, or even the co-working spaces shared with other companies or independent professionals. The driving force that selects the employees of a firm and manages most workplace initiatives, procedures, and even the culture of the firm is the HRM. This specific part of the firm creates the essence of the company, attracts new talent for the firm, implements training, and intends to assure the most effective and efficient working environment to achieve the goals of the organization, among other functions.

To address the current situation on HR practices, Table 1 showcases Forbes’ annual Top 10 HR trends for 2020 (Meister, 2020). In the year 2020, most trends move toward a better working environment, work-life balance, and skills. More and more firms are comprising resources to take care and to motivate their internal clients, their employees.

**TABLE 1:** Top HR talent attraction and retention trends 2020.

Position	2020
#1	Start with focusing on worker wellbeing
#2	Prepare for humans + bots as the new blended workforce
#3	Look for new use cases of AI 4 HR
#4	Focus on building ethical AI
#5	Consider soft skills to be power skills in 2020
#6	Audit your workplace environment for physical, emotional, and environmental attributes
#7	Explore virtual reality for corporate training
#8	Re-define blended learning to include on demand coaching
#9	Recruit for skills rather than college pedigree
#10	Make your workplace experience a top priority

*Source: Adapted from Meister (2020).*

As the new generation enters the labor market, HRM needs to take into consideration, and adapt to the previously mentioned characteristics of this cohort. This does not only mean attracting the Gen Z employees in a different way and offer them a variety of work-related benefits to draw them to and keep them in the organization, but also to redefine entry-level jobs (O’Boyle et al., 2017). There are only few research papers on how the labor market adapts to the needs and expectations of the Gen Z cohort, existing the need of further research. This may be because the majority of Gen Z individuals have been studying until lately and is only now starting to enter into the labor market.

Some articles studied the relationship between of employees, and companies or positions. The first one would be a person-organization fit model, so that the characteristics of the companies are congruent with the needs and wants of their employees (Graczyk-Kucharska and Erickson, 2020). The second would be an employee-job fit, with the aim of attaining a job satisfaction, as well as the work engagement and performance (Truxillo et al., 2012).

According to Bielen and Kubiczek (2020), the most common way the businesses respond to the demands of Gen Zers are friendly working atmosphere, keeping up with the latest technologies, ambassador programs, internships, benefits, and corporate social responsibility activities. Similar ideas are reported by Randstad Canada (2014). However, to be able to do so, organizations need to have the courage to break traditional approaches by using the existing tools in different ways, accepting that even individuals from the same generation and their needs may differ from their cohort prototype (O’Boyle et al., 2017), like what many firms have done with the on-line recruitment as their initial step (Tato-Jimenez et al., 2019). Additionally, HR departments should be preparing to introduce or to change workplace values and culture among other aspects (Graczyk-Kucharska and Erickson, 2020), rather than expecting Gen Z to adapt to the company.

Now that Gen Zers enter into the workforce, some organizations will have four or even five different generations working together. As Urick (2019, p. 78) states, generational differences in the workplace can lead to “intergenerational biases, stereotypes, and misperceptions,” and create conflict situations. This said, it is safe to assure that different generational cohorts with their own work preferences should have distinguishing job characteristics (Hernaus and Poloski Vokic, 2014). As previously done with Generation Y, companies need to modify their dynamics in order to manage the intergenerational diversity faced. To tackle these challenges, HRM has to explore new ways of satisfying the newcomers at the same time as trying not to neglect the employees of older generations, and learning how to manage multigenerational teams.

## Methods

This article pretends to bring to light the research trends involving Gen Z, the workplace, and the professional relations of this generation. For this purpose, a bibliometric review has been elaborated by collecting data from the Clarivate Analytics Web of Science (WoS), which has later been analyzed using the SciMAT open-source science mapping software tool.

### Search Strategy

The documents collected for this review have been retrieved from the WoS database as of October 14, 2021. When conducting the search for the three variants of Gen Z keywords, as well as “workplace,” “workforce,” and “employee” were used within the topic field, creating the advanced search algorithm in WoS: TS = (“Generation Z” OR “Gen Z” OR “Z Generation”) AND TS = (“workforce*” OR “workplace*” OR “employee*”).

To obtain the widest range of results due to the early stages of research on Gen Z, all the years (1900–2020) and the languages were accounted for, and the document type was not limited, thus, including reviews and conference proceeding papers among others. Additionally, the citation indexes selected were as follows: Science Citation Index Expanded (SCI-EXPANDED), Social Sciences Citation Index (SSCI), Arts & Humanities Citation Index (A&HCI), Conference Proceedings Citation Index- Science (CPCI-S), Conference Proceedings Citation Index- Social Science & Humanities (CPCI-SSH), Book Citation Index– Science (BKCI-S), Book Citation Index– Social Sciences & Humanities (BKCI-SSH), and Emerging Sources Citation Index (ESCI).

### Sample

The search completed on WoS obtained a total of 102 results. The analysis covers a 12-year time period from 2009 to 2020, and even if the WoS search was conducted from 1900, Gen Z is a much more recent term. Hence, to develop a longitudinal analysis, the literature has been divided into three periods. The expanse of these periods and their segmentation has been defined following a quantitative criterion of the number of the documents published, trying to find the most homogeneous stages possible (Table 2).

**TABLE 2:** Periods and documents per period.

Period number	Period	Number of documents
1	2009–2017	19
2	2018	22
3	2019	32
4	2020	29

*Source: Own elaboration from SciMAT data.*

The first stage covers documents from 2009 to 2017, the second corresponds with 2018, the third refers to 2019, and the last and fourth is 2020. Thus, considering the scant research until 2017 included, the first period has less manuscripts than the latter three, including only 19 publications in WoS from 2009 to 2017, from which five correspond to the last year of the stage. On the other hand, the 2018 period englobes a total of 22 papers, the third period, 2019, includes 32 articles, and, finally, 2020 addresses 29 registered documents in the database.

### Software

To be able to elaborate and to detangle the bibliometric analysis, SciMAT, an open-source science mapping software tool has been utilized. The reasons behind the decision to use this tool lie in the benefits it supplies the researcher. The SciMAT, created by Cobo et al. (2012), offers methods, measures, and algorithms for the whole general science mapping workflow, for which researchers usually need to apply various software tools. On this note, the SciMAT software allows the pre-processing of the data retrieved from WoS, Scopus, PubMed, or similar, for a posterior network extraction, the application of different normalization measures, mapping, and analysis, and the graphical visualization of the results (Cobo et al., 2012). The wizard analysis of the software allows to see a longitudinal map, which is one of the principal objectives of this article, as well as strategic maps and thematic networks.

**FIGURE 1:**
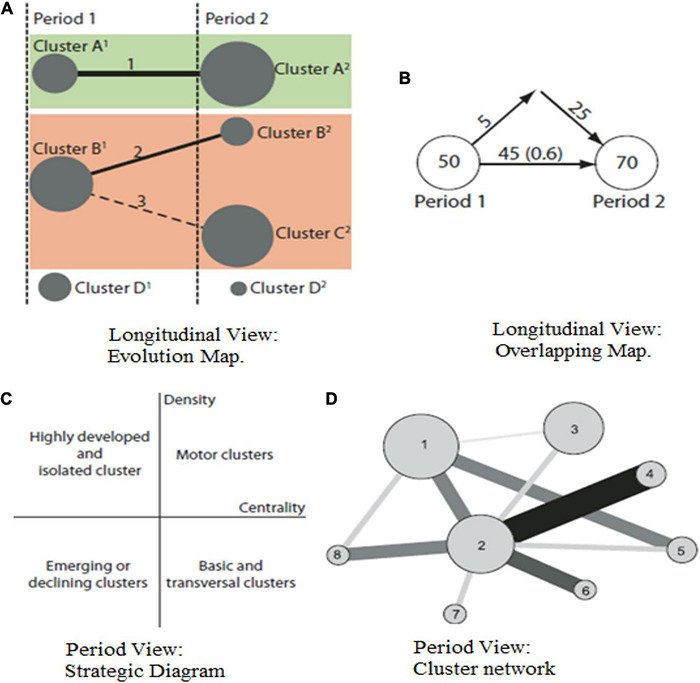
Evolution view and period view. Source: Cobo et al. (2012).

Within the longitudinal view, the evolution map (Figure 1A, left) shows on columns the different periods of the sample, showing the most relevant in clusters. These clusters are connected throughout the periods by lines, which represent the timely evolution of the themes. If two clusters are linked by a continuous line, these share the main item; but, if between two clusters, there is a discontinuous link meaning that they share elements but not the main item. Some may not be connected by lines, and in that case and if not appearing in the next period, the cluster has disappeared; if it suddenly appeared in a later period, the cluster is considered a new one. The size of each cluster depends on the selected performance measures. In the case of our study, we are considering the number of documents, the number of citations and the average citations, as well as the h-index, all with respect to the period and cluster chosen.

Additionally, an overlapping map (Figure 1B, right) represents the periods, and the number of keywords, in our case, each period is associated to. The upper outgoing arrow represents the keywords that have disappeared from one period to the next one, and the upper incoming arrows indicate the keywords added to the new period. The arrows connecting the periods offer the number of keywords shared among them, including the Stability Index between them.

The period view allows the user to decide which period is shown in the strategic diagram, and to choose the theme displayed in the cluster’s network. The SciMAT software wizard provides by defect the Callon’s density (Callon et al., 1991) and centrality measures. A strategic map (Figure 1C, left) showcases the most important themes of a given period, distributes in the figure according to their density and centrality ranges. This two-dimensional map divides the clusters into the following: motor clusters, being the ones with highest density and centrality; highly developed and isolated clusters or peripheral themes, with high density but low centrality; basic and transversal clusters when they have a high centrality but a low density; and emerging or declining clusters with both low density and centrality. For each cluster represented in the strategic map, a cluster network (Figure 1D, right) is provided with the related themes.

## Results

### Activity

**FIGURE 2:**
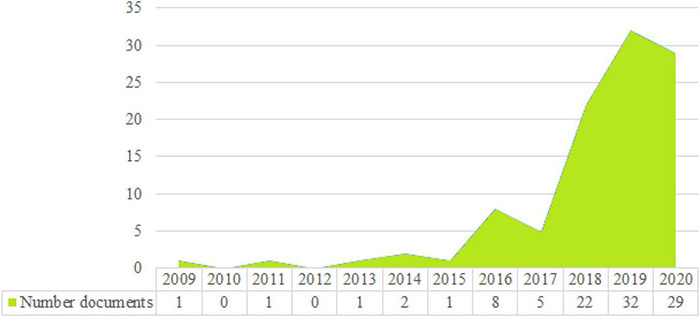
Number of publication per year. Source: Own elaboration from SciMAT data.

The WoS sample was composed by 102 documents published between 2009 and 2020 (Figure 2). It can be noticed that there are two distinct trends according to the rate of annual publications. In the time frame from 2009 to 2017, there is little research on Gen Z *per se*, and even less on their preparation, perceptions, and implications on the labor market, possibly because Gen Zers were mostly 22 years old and only a minority was working, while most of them were studying and other were just being born in 2009 and 2010. A second trend can be appreciated since 2018, from which Gen Z has been gaining relevance in research, as not only this generation starts working, but begins to have a perfect age for investigators to get an insight about their characteristics, preferences, values, and attitudes.

The 102 documents forming the analysis sample were written by a total of 234 published authors. From these authors, only Goh stands out from the rest, having written four articles. The low number of works on the topic is accompanied by the scarce research on Gen Z and the workforce, as well as the workplace of each author.

The most cited article is the work of Goh. It focuses on the hospitality sector and on Gen Z based on the theory of the planned behavior (Goh and Kong, 2018; Goh and Lee, 2018). Besides, the Gen Z motivations of the employees in the hospitality industry toward the food waste (Goh and Jie, 2019), the talent management and the recruitment strategies (Goh and Okumus, 2020) are the next more cited articles. The rest of authors have been involved in 1 or 2 papers each, suggesting that this topic is not their main line of research. We can also highlight the citations received in WoS by each analyzed author. Displayed in Table 3 are the authors with more than 20 citations.

**TABLE 3:** Authors with more than 20 citations ordered by authors.

Article’s title	Author/s	Year	Cites
The Employees of Baby Boomers Generation, Generation X, Generation Y and Generation Z in Selected Czech Corporations as Conceivers of Development and Competitiveness in their Corporation	Bejtkovsky, 2016	2016	22
Y and Z Generations at Workplaces	Bencsik et al., 2016	2016	75
Connecting with Generation Z: Approaches in Nursing Education	Chicca and Shellenbarger, 2018	2018	47
To waste or not to waste: Exploring motivational factors of Generation Z hospitality employees toward food wastage in the hospitality industry	Goh and Jie, 2019	2019	38
Theft in the hotel workplace: Exploring frontline employees’ perceptions towards hotel employee theft	Goh and Kong, 2018	2018	21
A workforce to be reckoned with: The emerging pivotal Generation Z hospitality workforce	Goh and Lee, 2018	2018	80
Avoiding the hospitality workforce bubble: Strategies to attract and retain generation Z talent in the hospitality workforce	Goh and Okumus, 2020	2020	29
The Changing Face of the Employees-Generation Z And their Perceptions of Work (A Study Applied to University Students)	Ozkan and Solmaz, 2015	2015	36
Are You Ready for Gen Z in the Workplace?	Schroth, 2019	2019	25

*Source: Own elaboration from SciMAT data.*

It can be seen how Goh has paved the way again with 92 citations from his three articles, each with the collaboration of one other author: Lee, Okumus, and Kong, were all included in Table 3. Within the publications of Goh, *A workforce to be reckoned with: The emerging pivotal Generation Z hospitality workforce* (Goh and Lee, 2018) is worth mentioning, as the article has received a significant number of citations within WoS, making Lee the second most cited author. Additionally, Bejtkovsky (2013, 2016) has contributed on two documents, dated in 2013 and 2016. However, 17 received citations corresponds only to one of his articles, *The Employees of Baby Boomers Generation, Generation X, Generation Y, and Generation Z in Selected Czech Corporations as Conceivers of Development and Competitiveness in their Corporation* (Bejtkovsky, 2016), which was dedicated to multiple generations within the workplace, generation gaps, and human resources.

The rest of the authors on the list have only one paper each, but as observed, have higher citations (more than 20 citations) than most top writers on our sample Table 3. Both Ozkan and Solmaz (2015) have received the third best citation number on their participation in the 4th World Conference On Business, Economics, And Management with The Changing Face of the Employees—*Generation Z and Their Perceptions of Work (A Study Applied to University Students)*. Apart from the abovementioned authors, Schroth wrote about the readiness of the workplace to receive Gen Z (Schroth, 2019), while both Lazanyi and Bilan (2017) collaborated to create an article on the trust of Gen Zers toward others within the workplace.

Additionally, the whole 102 document sample is associated with 91 journals. Table 4 shows the most influential journals according to the 2020 journal impact factor (JIF) provided in WoS, the corresponding quartile on the Journal Citation Report (JCR), the number of documents published on the topic, and the number of citations. Table 4 only includes those journals with more than 20 citations. The JIF is the ratio obtained by dividing the number of citations of a journal on the previous two years by the number of articles published by the journal over the same time period. In the case of this study, the 2019 JIF has been used, being the number of citations of the specific journal in 2018 and 2017 divided by the total of the published documents in it.

**TABLE 4:** Journals with most impact.

Article’s title	Year	JIF	Quartile	Author/s	Citations
International Journal of Hospitality Management	2019	6.701	Q1	Goh and Jie, 2019	38
International Journal of Hospitality Management	2018	4.465	Q1	Goh and Lee, 2018	80
California Management Review	2019	3.909	Q2	Schroth, 2019	25
Journal of Competitiveness	2016	3.649	Q2	Bejtkovsky, 2016	22
Journal of Competitiveness	2016	3.649	Q2	Bencsik et al., 2016	75
Tourism and Management Perspectives	2020	3.648	Q2	Goh and Okumus, 2020	29
Sustainability	2020	3.251	Q2	Cresnar and Nedelko, 2020	8
Sustainability	2020	3.251	Q2	Smaliukiene and Bekesiene, 2020	5
International Journal of Environmental Research and Public Health	2019	2.849	Q2	Sánchez-Hernández et al., 2019	7
Sustainability	2018	2.592	Q2	Cho et al., 2018	14
Sustainability	2019	2.576	Q2	Tato-Jimenez et al., 2019	0
Sustainability	2019	2.576	Q2	Gyurak Babelova et al., 2019	4
Frontiers in Psychology	2019	2.067	Q2	Sobrino-De Toro et al., 2019	1
Scandinavian Journal of Psychology	2021*	1.570	Q2	Mahmoud et al., 2021	5
International Journal of Management Education	2019	2.354	Q3	Maloni et al., 2019	6
Journal of Nursing Management	2018	2.243	Q3	Christensen et al., 2018	13
Anales de Psicologia	2020	1.346	Q3	Zúñiga Ortega et al., 2019	1
Journal of Nursing Administration	2019	1.274	Q3	Hampton and Welsh, 2019	6
Transformations in Business & Economics	2020	1.621	Q4	Snieska et al., 2020	4
Journal of Organizational Change Management	2020	0.967	Q4	Chillakuri, 2020	7
International Journal of Manpower	2021*	0.953	Q4	Mahmoud et al., 2021	9
Journal of Business-to-Business Marketing	2019	0.543	Q4	Rodriguez et al., 2019	14

*Source: Own elaboration from SciMAT data and 2019 Journal Citation Reports.WoS has classified included into year 2020.*

Concerning the WoS categories, the three most frequent category is *Management*; followed by *Hospitality and Tourism, Business*, and *Economics*. During the analyzed period (2009–2020) there are 15 different management journals, five hospitality and tourism journals, and four business and economics. The journals with major impact—considering the articles with more than 20 citations—are the *International Journal of Hospitality Management*, the *California Management Review*, the *Journal of Competitiveness*, and the *Tourism and Management Perspectives*. Only the former journal is in the first quartile and has a JIF that is remarkably higher than the rest.

However, in general terms, the highest number of articles concerning this theme (5) have been published by the journal *Sustainability* in the years 2018 (JIF 2.592), 2019 (JIF 2.576), and 2020 (JIF 3.251). This was followed by the *Journal of Competitiveness* in the year 2016 (JIF 3.649), and *International Journal of Hospitality Management* (2) in the years 2018 (4.465) and 2019 (JIF 6.701).

### Evolution of Keywords

**FIGURE 3:**
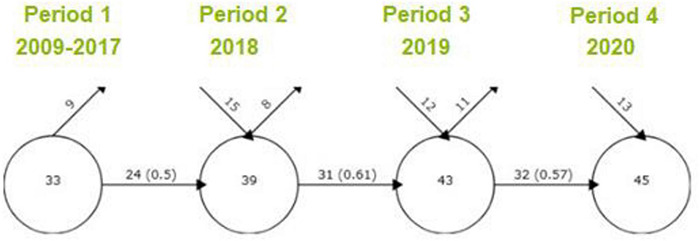
Keywords between periods. Source: Own elaboration with SciMAT.

Now, the evolution of the keywords along the different periods will be addressed (Figure 3). The first period (2009–2017), although aggregating an eight-year time period, is characterized by a lower number of keywords than the rest of periods, which actually have a very similar amount. In the first period, there were 33 keywords, from which, nine were no longer used in the following ones. More than half of the words used (0.5) in this period were also transferred to the second one. For the second period, 15 additional words were introduced, summing up to 39 total keywords. Again, eight keywords disappeared and 31 transitioned to the next period, representing almost three fourths (0.61) of the total word count in 2019, being higher than the common keyword proportion of the change from period 1 to period 2. In the third period, 12 keywords were included in the topic research, adding up to 43 total keywords. Then, 32 keywords were included in the last period and 11 keywords were lost and will not be used in the following period. In the last period 13 words were included in the research of the topic, totaling of 45 keywords. The difference between the number of keywords between the first and the last periods is relatively low but can be justified by the scarce research on the topic. These results suggest that there is a wide development margin in the literature relating to Gen Z within the workforce and the workplace.

### Longitudinal Analysis

**FIGURE 4:**
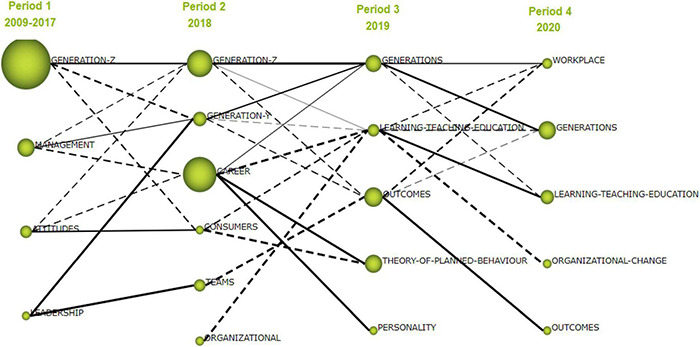
Theme evolution of primary documents. Source: Elaborated with SciMAT.

By means of the longitudinal diagram (Figure 4) provided by SciMAT, the current evolution of the research related to the topic of this article will be analyzed. Some clusters have been maintained during two periods or have disappeared and then reappeared in a later period. It should be reminded, that each cluster was selected to have a maximum of 10 items and a minimum of 2.

With eyes on the first period, from 2009 to 2017, it can be seen how there are only four clusters: “Generation-Z,” “management,” “attitudes,” and “leadership.” The reason for the scarce number of clusters in this period is due to two facts: the keywords of the sample are focused on those concrete clusters—there is a larger number of keywords, but were not grouped in clusters due to the minimum requisite of two items and there is a small number of publications. For our study, clustering through an algorithm of the simple center with a minimum network size of 2 is important, because otherwise there would be an excessively large number of clusters. This is due to the high number of keywords of different thematic, as the research on this area was not developed enough during this period.

For the second period, year 2018, there are six clusters: “Generation-Z,” “Generation-Y,” “career,” “consumers,” “teams,” and “organizational.” The “Generation-Z” cluster is the only one maintained from the 2009–2017 period, gaining relevance within the research made during 2018 because it is placed in a better location in the strategic diagram, with higher centrality and density values, being the motor theme of this period. This was materialized in articles such as “*A workforce to be reckoned with: The emerging pivotal Generation Z hospitality workforce*” (Goh and Lee, 2018), or “*Generation Z’s Sustainable Volunteering: Motivations, Attitudes and Job Performance*” (Cho et al., 2018). The “Generation-Y” cluster has a linkage with the previous “management” and “leadership” clusters. Additionally, the leadership cluster (2018) has also a strong and direct relationship with the cluster “teams” from the first period. The “career” cluster is the most relevant by grouping documents based on the number of citations. However, since the density and the centrality of the career do not have a defined role in this theme yet, its evolution in the following periods remains uncertain.

In the 2019 period all the clusters are new. Outstanding for the number of citations are “outcomes,” followed by “Theory of planned behavior,” which is connected to the previous period through “career.” Likewise, “generations” is directly related to the “Generation-Z” cluster. An example of how the intergenerational view gains strength is the article *Critical elements for multigenerational teams: a systematic review* (Burton et al., 2019). Other clusters in this period are “learning-teaching-education” and “personality” which has a linkage with the second period cluster “career.”

In the 2020 period, various clusters from the previous period are consolidated. The case of “generations,” “learning-peaching-education” or “outcomes” have a very strong linkage with the same clusters of the previous period. “Organizational change” is the weakest cluster. While “workplace” appears strongly as it is placed as the driving theme of the last period and has a direct linkage with the most important clusters of the rest of the periods. The article entitled *Understanding Future Leaders: How Are Personal Values of Generations Y and Z Tailored to Leadership in Industry 4.0?* (Cresnar and Nedelko, 2020) is an example.

Following the analysis of the longitudinal diagram and considering the aforementioned remarks on the evolution of the words, a quantitative comparison has been elaborated from the data provided by SciMAT. Table 5 shows the clusters of each period of the sample, the centrality and density of each, as well as the number of documents, the number of citations, and the average citations within the theme, ending with the h-index provided by SciMAT. Within the first period (2009–2017), the cluster “Generation-Z” needs to be highlighted due to its high impact in terms of number of citations (171 citations), as well as the documents published (13 documents). Accordingly, “attitudes” stands out as the cluster with the highest average number of citations (15 citations) achieved with a single document. Also, “management” is one of the clusters with less centrality than “Generation-Z” but has a higher density, and, therefore, has a prominent position among the driving themes of this period (Table 5).

**TABLE 5:** Quantitative factors of the themes and their evolution.

	Centrality	Density	Documents	Citations	Average citations	H-Index
**2009–2017**						
Generation-Z	103.69	52.48	13	171	13.15	5
Management	55.9	87.5	5	38	7,6	3
Attitudes	0	0.25	1	15	15	1
Leadership	15.82	50	1	0	0	0
**2018**						
Generation-Z	145.61	54.06	8	73	9.125	3
Generation-Y	51.85	51.94	7	23	3.29	2
Career	37.01	46.3	4	105	26.25	3
Consumers	10	100	1	0	0	0
Teams	11.75	50	1	14	14	1
Organizational	52.22	50	1	2	2	1
**2019**						
Generations	92.12	52.16	13	33	2.54	3
Learning-Teaching-Education	74.36	24.12	15	15	1	2
Outcomes	43.81	69.1	4	43	10.75	3
Theory of planned behavior	12.58	50	1	38	38	1
Personality	3.86	25	1	0	0	0
**2020**						
Workplace	74.48	67.36	4	8	2	1
Generations	165.25	48.18	13	38	2.92	4
Learning-Teaching-Education	106.25	22.39	9	22	2.44	3
Organizational Change	10.95	50	1	2	2	1
Outcomes	30	50	1	0	0	0

*Source: Obtained from SciMAT.*

When considering the second period (2018), “career” is the highlighted cluster of the year. Similarly, to “Generation-Z” in the first period, this cluster manages to draw the attention for the highest number of citations (105 citations), being the higher on average citations (26.25 cites). The second with most cited in this period is “Generation-Z” with 73 citations. Also, “Generation-y” has 23 citations and an average citation of 3.29 per article. The cluster “teams” with only one published article involves a relevant impact as it reaches 14 citations in comparison with clusters of “innovations” and “consumers” with two and cero citations, respectively.

The third period (2019) concentrates the clusters of current trends. Nowadays, the most researched themes are, in descending order of citations: “outcomes” with 43 citations, “theory of planned behavior” with 38 citations, “generations” with 33 citations, “learning-teaching-education” with 15 citations, and “personality” cluster has no citation.

The fourth and last period (2020) is a continuation of the previous period in terms of clusters highlighted by number of citations. “Generations” and “learning-teaching-education” stood out by the cited papers, with 38 and 22 citations, respectively, followed by “workplace” with eight citations. Although this last cluster has a lower number of citations, it should be noted that only four articles have been cited, so the average number of citations is very close to the first two clusters.

From a general perspective, there are some observations to consider. (a) The clusters created in the first period (“Generation-Z,” “management,” “attitudes,” and “leadership”) have served as a base for the research of the forthcoming years, have been a major impact, particularly “Generation-Z” as they have transitioned to another period. (b) The number of total documents of the clusters seem to increase with time. (c) Notably, the clusters with most impact during the whole timeframe of the analysis, which are from higher to lower number of citations, are as follows: “Generation-Z” (171 citations), “career” (105 citations), “outcomes” (43 citations), and “generations” (38 citations).

### Period-by-Period Strategy Map Analysis

Once the longitudinal map has been explained, and the evolution of the clusters is known, the paper will proceed analyzing the importance of each cluster in terms of Callon’s density and centrality measures through a strategic map, which values are represented and already mentioned in Table 5. Centrality measure of Callon represents the interaction among networks, whereas the density measure indicates the internal strength of the network. After the analysis of a strategic diagram of the period, the main cluster in the motor themes is addressed, meaning, the theme that has the highest combination of density and centrality is the one cluster that is in the most upper-right position in the map. The analysis of the network surrounding the motor theme of each period is then explained.

#### First Period 2009–2017

**FIGURE 5:**
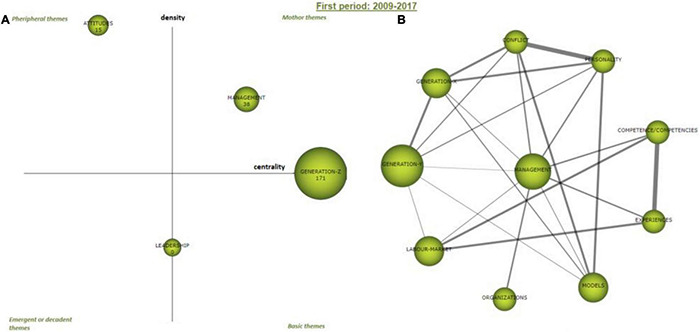
Period 2009–2017 strategic diagram **(A)** and the motor cluster’s network **(B)**. Source: Elaborated with SciMAT.

The strategic map (Figure 5) visually shows how the clusters of the 2009–2017 period are scattered according to their density and centrality measures (previous Table 5). The cluster “Generation-Z” is a relevant cluster which, although has the higher number of articles, the early stages of the topic in this period makes its average citation lower than would be expected. “Generation-Z” stands with a centrality of 103.69 and a density of 52.48. On this note, “Generation-Z” is an important topic in the research, but this needs additional development. The “management” cluster is a driving theme due to its measures on centrality (55.9) and density (87.5). Thus, the “management” network has high interaction and internal strength and was at the center of the research in this period.

On the other hand, we have “leadership,” which is between an emergent or a decadent theme and a motor theme, with 15.85 and 50 centrality and density values, respectively. As the topic of this study is quite recent and does not amount to excessive research, a focus on the “leadership” in so early stages makes it highly interesting as the same time than the specialized theme. Changing quadrant, the cluster “attitudes” is clearly peripheral theme, with a centrality of 0 and a density of 0.25. This last cluster will disappear on the rest of periods (2018, 2019, and 2020).

Now, the thematic network of the motor theme (Figure 5, right) of the period will be analyzed, driving the internal analysis of the “management” cluster. In order to disclose insights on the most relevant links, the weight of the internal links is displayed in Table 6.

**TABLE 6:** “Generation-Z” and Management cluster network 2009–2017.

Node A	Node B	Weight
GENERATIONS	BABY-BOOMERS	0.25
GENERATIONS	WORKPLACE	0.33
GENERATIONS	TRADITIONALISTS	0.25
GENERATIONS	GENERATION-Z	0.09
GENERATIONS	HUMAN-RESOURCES	0.25
BABY-BOOMERS	WORKPLACE	0.33
BABY-BOOMERS	TRADITIONALISTS	1
BABY-BOOMERS	GENERATION-Z	0.09
BABY-BOOMERS	HUMAN-RESOURCES	0.25
WORKPLACE	TRADITIONALISTS	0.33
WORKPLACE	GENERATION-Z	0.12
WORKPLACE	DIGITAL	0.08
WORKPLACE	HUMAN-RESOURCES	0.75
SERVICES	GENERATION-Z	0.09
ORGANIZATIONAL-CHANGE	GENERATION-Z	0.09
ORGANIZATIONAL-CHANGE	DIGITAL	0.25
TRADITIONALISTS	GENERATION-Z	0.09
TRADITIONALISTS	HUMAN-RESOURCES	0.25
GENERATION-Z	DIGITAL	0.36
GENERATION-Z	EMOTIONS	0.09
GENERATION-Z	LEARNING-TEACHING-EDUCATION	0.16
GENERATION-Z	EMPLOYMENT	0.09
GENERATION-Z	HUMAN-RESOURCES	0.2
DIGITAL	LEARNING-TEACHING-EDUCATION	0.05
DIGITAL	HUMAN-RESOURCES	0.06
EMOTIONS	EMPLOYMENT	0.25
LEARNING-TEACHING-EDUCATION	EMPLOYMENT	0.05
EMPLOYMENT	HUMAN-RESOURCES	0.06

*Source: Elaborated from SciMAT data.*

The network around the main themes of the period is composed by the following internal links: (a) “experiences” is linked with “competence,” and (b) “conflict” with “personality.” To a lesser extent, latter clusters are also connected with “Generation-Y” and “Generation-X,” the “experiences” cluster with “labor market,” and the latter with “Competence.” The different models to be adopted in terms of conflict and the personality of workers are also part of the network of this main theme.

#### Second Period 2018

**FIGURE 6:**
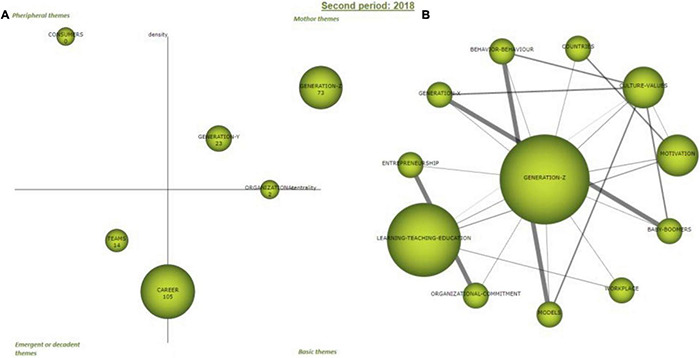
Period 2018 strategic diagram **(A)** and motor cluster’s network **(B)**. Source: Elaborated with SciMAT.

Likewise, strategic map of the 2018 period (Figure 6, left) visually shows how the clusters of the period are distributed according to density and centrality measures (Table 5), increasing the number of clusters by one with respect to the previous period.

In this case, there are two motor clusters that define the thematic of the period, “Generation-Z” and “Generation-Y.” Both clusters attract publications and have high average citations. The “Generation-Z” cluster is characterized by a high interaction (145.61) and by internal strength (54.06). In the Figure 6, it is closer to the top right corner of the strategic diagram, meaning, it is the most influential thematic in the period. The “Generation-Y” cluster is also a motor theme but to a lesser extent, with centrality of 51.85 and a density of 51.94.

There are two groups of clusters on the border of the basic topics-emergent and decadent themes and on the border of basic topics-motor themes. On the one hand, “career” is the cluster that receives the highest number of citations, but due to its density, it is on the borderline between the emerging or decadent themes and the basic themes. In turn, the “organizational” cluster, due to its centrality, is on the borderline between basic and driving themes. We will have to check their evolution to see if they will finally fall into one of the surrounding quadrants.

Within the peripheral themes, “consumers” is a new cluster. Finally, the “teams” cluster can classify as an emergent or decadent theme, not very developed, with a centrality of 11.75, and the lowest density within the sample (50).

Regarding the internal thematic analysis of the main motor theme network (Figure 6, right and Table 7), “Generation-Z” maintains the most important links as follows: “Baby-boomers” with “Generation-X”; “models” with “behavior”; and “organizational commitment” with “Entrepreneurship.” Moreover, to a lesser extent, “culture and values” with “Generation-X,” “behavior,” “models,” and “baby-boomers.”

**TABLE 7:** “Generation-Z” Cluster Network 2018.

Node A	Node B	Weight
BABY-BOOMERS	GENERATION-Z	0.11
BABY-BOOMERS	GENERATION-X	1
BABY-BOOMERS	CULTURE-VALUES	0.33
WORKPLACE	GENERATION-Z	0.11
WORKPLACE	LEARNING-TEACHING-EDUCATION	0.14
MODELS	GENERATION-Z	0.11
MODELS	BEHAVIOR-BEHAVIOUR	1
MODELS	CULTURE-VALUES	0.33
ORGANIZATIONAL-COMMITMENT	GENERATION-Z	0.11
ORGANIZATIONAL-COMMITMENT	ENTREPRENEURSHIP	1
GENERATION-Z	LEARNING-TEACHING-EDUCATION	0.14
GENERATION-Z	ENTREPRENEURSHIP	0.11
GENERATION-Z	GENERATION-X	0.11
GENERATION-Z	BEHAVIOR-BEHAVIOUR	0.11
GENERATION-Z	COUNTRIES	0.11
GENERATION-Z	CULTURE-VALUES	0.15
GENERATION-Z	MOTIVATION	0.15
LEARNING-TEACHING-EDUCATION	CULTURE-VALUES	0.05
LEARNING-TEACHING-EDUCATION	MOTIVATION	0.19
GENERATION-X	CULTURE-VALUES	0.33
BEHAVIOR-BEHAVIOUR	CULTURE-VALUES	0.33
COUNTRIES	MOTIVATION	0.33
CULTURE-VALUES	MOTIVATION	0.11

*Source: Elaborated from SciMAT data.*

#### Third Period 2019

**FIGURE 7:**
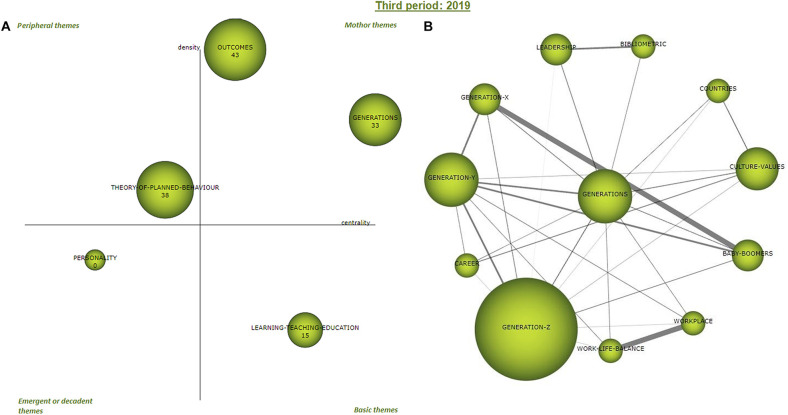
Period 2019 strategic diagram **(A)** and motor cluster’s network **(B)**. Source: elaborated with SciMAT.

The strategic map in Figure 7 (left) shows how the cluster of 2019 period stands according to density and centrality measures of Callon (previous Table 5). There are two driving themes that define the thematic of the period and attract publications, “outcomes” and “Generation-Z,” which define the current tendency of the published articles. Both are characterized by the highest internal strength of their networks, being 82.90 and 81.21, respectively. One of the main differences between these two motor themes is that “Generation-Z” has attracted far more publications (22) and citations (46 cites) than “impact” (numbers 6 and 7). The “Generation-Z” cluster appeared in the first period (2009–2017), losing importance in the second (2019) as it did not even appear in the longitudinal map, and reappearing even more strongly in the last one (2020–2021). In this new appearance of “Generation-Z”, its centrality increased at over 50% and its density at over 60%.

The cluster “personality” enters into play as an emergent theme due to a low centrality and “learning-teaching-education” as a basic theme with a higher centrality being its internal consistency more important in comparison to “personality” (Figure 7, left).

Additionally, there is one peripheral theme, “theory of planned behaviour” which has evolved from the second period (2018) from the cluster’s “career” and “consumers.”

With regards to the internal thematic analysis of “generations” as the main driving theme network of the period (Figure 7, right), the internal links are shown in Table 8. In this specific period, the relations with the highest density are: “workplace” with “Generation-Z”; and “baby-boomers” with “Generation-X.” To a lesser extent is observed the linkage of “Generation-Y” with “Generation-Z,” and “baby-boomers” with “Generation-X.” All clusters share links to other themes with lower weights.

**TABLE 8:** “Generations” cluster network 2019.

Node A	Node B	Weight
GENERATIONS	BABY-BOOMERS	0.15
GENERATIONS	WORKPLACE	0.11
GENERATIONS	WORK-LIFE-BALANCE	0.11
GENERATIONS	GENERATION-Z	0.18
GENERATIONS	CAREER	0.11
GENERATIONS	GENERATION-Y	0.31
GENERATIONS	GENERATION-X	0.15
GENERATIONS	LEADERSHIP	0.15
GENERATIONS	BIBLIOMETRIC	0.11
GENERATIONS	COUNTRIES	0.11
GENERATIONS	CULTURE-VALUES	0.17
BABY-BOOMERS	GENERATION-Z	0.14
BABY-BOOMERS	GENERATION-Y	0.33
BABY-BOOMERS	GENERATION-X	1
WORKPLACE	WORK-LIFE-BALANCE	1
WORKPLACE	GENERATION-Z	0.05
WORKPLACE	GENERATION-Y	0.11
WORK-LIFE-BALANCE	GENERATION-Z	0.05
WORK-LIFE-BALANCE	GENERATION-Y	0.11
GENERATION-Z	CAREER	0.05
GENERATION-Z	GENERATION-Y	0.32
GENERATION-Z	GENERATION-X	0.14
GENERATION-Z	LEADERSHIP	0.02
GENERATION-Z	COUNTRIES	0.05
GENERATION-Z	CULTURE-VALUES	0.07
CAREER	GENERATION-Y	0.11
CAREER	CULTURE-VALUES	0.17
GENERATION-Y	GENERATION-X	0.33
GENERATION-Y	CULTURE-VALUES	0.07
LEADERSHIP	BIBLIOMETRIC	0.33
COUNTRIES	CULTURE-VALUES	0.17

*Source: Elaborated from SciMAT data.*

#### Fourth Period 2020

**FIGURE 8:**
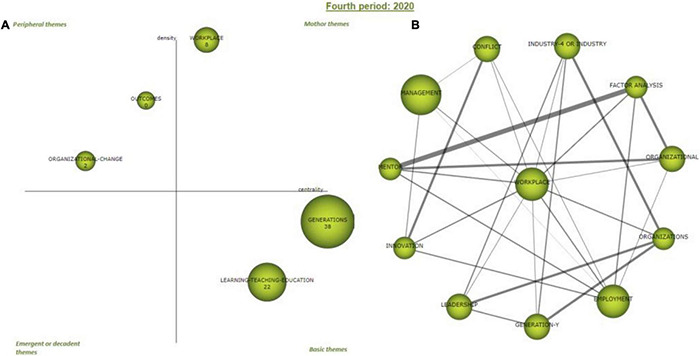
Period 2020 strategic diagram **(A)** and motor cluster’s network **(B)**. Source: elaborated with SciMAT.

In this last period, the “workplace” cluster appears as a driving theme, leading the research in the last year 2020. If we consider the evolution of the themes in the longitudinal analysis (Figure 8), the “workplace” cluster is linked to the “Generation-Z” and “generations” clusters of previous periods. In this last period, it reaches the necessary internal consistency to be the leading researching theme. On the other hand, the “generations” cluster remains as a basic theme without evolving with respect to 2019, although it receives 22 citations in 2020. The peripheral themes are “organizational change” and “outcomes,” losing its relevance from 2019, although it receives 22 citations in 2020. The peripheral themes are “organizational change” and “Outcomes” which in the previous period was a driving theme, and also, they lost their relevance in 2020. There are no emerging or declining themes for this period.

Regarding the internal analysis of the driving theme “workplace” (the main thematic network driving the period) (Figure 8, right), the internal links are shown in Table 9. The most intense relationships are: “mentor” and “factor analysis” and to a lesser extent “organizations” and “industry 4.0,” “organizations” and “leadership,” “organizations” and “Generation-Y,” and “factor analysis” and “organizational.” Finally, from a cross-period approach, it is evident that the main driving theme is “Generation-Z” but obviously the keyword filter included in the search is the main reason.

**TABLE 9:** “Workplace” cluster network 2020.

Node A	Node B	Weight
WORKPLACE	ORGANIZATIONS	0.25
WORKPLACE	EMPLOYMENT	0.25
WORKPLACE	GENERATION-Y	0.12
WORKPLACE	LEADERSHIP	0.12
WORKPLACE	INNOVATION	0.25
WORKPLACE	MENTOR	0.25
WORKPLACE	MANAGEMENT	0.17
WORKPLACE	CONFLICT	0.12
WORKPLACE	INDUSTRY-4 OR INDUSTRY	0.12
WORKPLACE	FACTOR ANALYSIS	0.25
WORKPLACE	ORGANIZATIONAL	0.12
ORGANIZATIONS	GENERATION-Y	0.5
ORGANIZATIONS	LEADERSHIP	0.5
ORGANIZATIONS	INDUSTRY-4 OR INDUSTRY	0.5
EMPLOYMENT	INNOVATION	0.25
EMPLOYMENT	MENTOR	0.25
EMPLOYMENT	MANAGEMENT	0.04
EMPLOYMENT	CONFLICT	0.12
EMPLOYMENT	FACTOR ANALYSIS	0.25
EMPLOYMENT	ORGANIZATIONAL	0.12
GENERATION-Y	LEADERSHIP	0.25
GENERATION-Y	INDUSTRY-4 OR INDUSTRY	0.25
LEADERSHIP	INDUSTRY-4 OR INDUSTRY	0.25
INNOVATION	MANAGEMENT	0.17
INNOVATION	CONFLICT	0.5
MENTOR	FACTOR ANALYSIS	1
MENTOR	ORGANIZATIONAL	0.5
MANAGEMENT	CONFLICT	0.08
FACTOR ANALYSIS	ORGANIZATIONAL	0.5

*Source: Elaborated from SciMAT data.*

## Discussion

### Implications

The aim of this work has been to disclose the thematic research trends and their evolution on the Gen Z as employees within the workforce and in the workplace. Additionally, the authors wanted to shed light to the themes that have not been sufficiently developed yet. The idea is that this research should not fully dive into the content, which was used for the research, but rather to see a full context on the matter. This is to serve as a support for further research. The effectiveness, as well as the relevance of the methodology, used is proven, and the data collected has been properly uncovered through a series of step, which pretended to go from the general study fields as the authors or journal, to more concrete aspects of the topic, such as the network of the most influential thematic clusters of each period.

Additionally, 13 authors stood out from the rest, having written more than one article, while 33 have received more than 20 citations. From these, one author is worth mentioning, Goh, who not only has written three articles while the rest have written two or less, but he has also received the maximum number of citations (92) for its research on the topic related with the hospitality sector, the theory of planned behavior, talent management, and recruitment strategies (Goh and Kong, 2018; Goh and Lee, 2018; Goh and Okumus, 2020). The most relevant journals with respect to the JIF are the *International Journal of Hospitality Management*, the *California Management Review*, the *Journal of Competitiveness*, the *Tourism and Management Perspectives*, and the *Sustainability*, from which all are above 3.500 JIF index. An expected result from the journal analysis is the focus on management in the most impactful journals. Another interesting finding from the research is the orientation on hospitality and tourism, psychology, and nursing as related areas of the study joining the main topic of this research. But there is a lack of studies related with other sectors as basic, at the same time than complex, as financial sectors, or customer service other than from the hospitality and tourism industry.

Mostly from the tendency change in 2018, the number of publications on the topic have increased, but still are relatively low. The results demonstrate an increasingly meaningful line of research on Gen Z within the workforce and the workplace, since 2018. These research results are mainly from the management field, remarking the importance, and impact this generation has on companies and their dynamics. The highest number of works were published in 2019, after which a small decrease in the number of documents occurred in 2020.

The evolution of the keywords between the periods had the following effects: (a) there has only been an increase of 12 words within the whole length of the periods studied; (b) throughout this complete time frame, 28 keywords were discarded; (c) whereas 40 were included. Furthermore, the longitudinal analysis has allowed to discover that the four clusters of the first period (“generation Z,” “management,” “attitudes,” and “leadership”) have been the basis for the evolution of the theme.

The “generation Z” cluster stands out in the first and the second period. This cluster evolves into the “generations” cluster in the third period, and into the “workplace” cluster in the last period. We believe that this fact indicates the interest of considering workplace as an ecosystem in which Gen Z must interact with other previous generations. The size of the “workplace” cluster indicates that this line of research may potentially expand in the near future.

### Limitations

This article is admitted being subject to certain limitations. Firstly, the sample used for the research is small and was only exclusively obtained from the Web of Science database. In this case, the study could be compared with similar bibliometric analyses on the matter involving different databases, e.g., Scopus. Secondly, the elimination of documents not directly related to the topic and the subjective clustering of words into word groups may offer slightly different results if another person replicates the review.

## Conclusion

The special characteristics and behaviors of the newest generation to enter the labor market make the management of Gen Z within the workplace and in the workforce a real challenge. It is not only necessary to acknowledge that changes are coming, but also imperative to start adapting now if it has not started yet. When a new age cohort enters the workforce, firms and employees face a modification of the work dynamic and company culture. Therefore, the role of human resources management is crucial for an effective onboarding and for the correct adaptation to the new normal workplace. The addition of another generation to the work environment will affect both the professional and the social context in which the employees develop their careers. No misunderstanding or wrongful generalization of methods and techniques should be done, as the strategic goals of a firm are individual, and the formal and cultural structures of the company need to be aligned with it for correct decision-making.

As for the results of this research, they allow a better understanding of thematic field of the Gen Z related to the workforce and in the workplace. The analysis revealed the development of the study on Gen Z, the workforce, and the workplace in a time frame of 11 years. From 2009 to 2017, the number of publications is relatively low, and it is from 2018 when the topic starts attracting higher attention. Most authors have only written one document, whereas a few have written two, and only one stood out for the number of publications and citations, Goh, who has written four articles and received 168 citations among all his works. In addition, the journals with most impact are the *International Journal of Hospitality Management*, the *California Management Review*, the *Journal of Competitiveness*, and the *Sustainability.*

There is a slow but sustained growth of research on the topic, together with a relatively small rate of keywords incorporated and a low stability among periods. This suggests a weakly increasing interest of researchers in the field, and a broad margin for future development. Moving on to the analysis of the thematic cluster evolution, the distribution of themes has been discussed, and the driving theme network of each period has been displayed. The main core of the research on Gen Z within the workforce and in the workplace has been redundantly developing around “Generation-Z, “workplace,” “generations,” “learning-teaching-education,” and “career.” With the volatile progress of the key clusters on the topic through the periods, it is not safe to say which themes will be surely included in the next years, but it seems that Gen Z will keep a strong importance, as well as the current basic clusters, which are related to performance and the workforce.

There are some suggestions in relation to areas with the need of future research due to the absence or to insufficient publications developed. On one hand, a technological aspect on the workplace could be addressed to shed some light on how companies need to prepare or are presently preparing for a digital evolution in the workplace motivated by the tech-savvy Gen Z. On the other hand, it would be interesting to study the knowledge and skills of generation Z as leaders and how they are transferred to future generations.

## Author Contributions

ES-T, EN-R, GB-G, and MB-M designed, performed, analyzed the research, wrote the manuscript, searched literature, analyzed, and verified the data of this article. All authors contributed to the article and approved the submitted version.

## Conflict of Interest

The authors declare that the research was conducted in the absence of any commercial or financial relationships that could be construed as a potential conflict of interest.

## Publisher’s Note

All claims expressed in this article are solely those of the authors and do not necessarily represent those of their affiliated organizations, or those of the publisher, the editors and the reviewers. Any product that may be evaluated in this article, or claim that may be made by its manufacturer, is not guaranteed or endorsed by the publisher.
