# Supplementary material for: Generation Z Within the Workforce and in the Workplace: A Bibliometric Analysis
Source: Front Psychol. 2022 Feb 1;12:736820. doi: 10.3389/fpsyg.2021.736820 (PMC8844017; doi:10.3389/fpsyg.2021.736820)
Supplement: Supplementary file 1 [file Table_1.docx]

**Appendix (Tables)**

**TABLE 1**

**TABLE 1 ꟾ** Top HR talent attraction and retention trends 2020 and 2021.

| Position | **2020** | **2021** |
| --- | --- | --- |
| #1 | Start with focusing on worker wellbeing | Make employee wellbeing a top business mandate |
| #2 | Prepare for humans + bots as the new blended workforce | Use the coronavirus pandemic to accelerate your workplace transformation |
| #3 | Look for new use cases of AI 4 HR | Invest in mental health as a must have rather than a nice-to-have benefit |
| #4 | Focus on building ethical AI | Re-engineer and transform corporate learning, the time is now |
| #5 | Consider soft skills to be power skills in 2020 | Provide internal talent mobility to attract, engage, and retain employees |
| #6 | Audit your workplace environment for physical, emotional, and environmental attributes | Solving business problems using people analytics |
| #7 | Explore virtual reality for corporate training | Prepare for the hybrid office of the future |
| #8 | Re-define blended learning to include on demand coaching | Expand employee experience and wellbeing resources to the entire family unit |
| #9 | Recruit for skills rather than college pedigree | Be holistic in creating a diverse, equitable, and inclusive workforce |
| #10 | Make your workplace experience a top priority | Anticipate new HR jobs of the future |

*Source: Adapted from Meister (2020, 2021).*

**TABLE 2**

**TABLE 2 ꟾ** Periods and documents per period.

| **Period number** | **Period** | **Number of documents** |
| --- | --- | --- |
| 1 | 2009-2018 | 17 |
| 2 | 2019 | 21 |
| 3 | 2020-2021 (May) | 26 |

*Source: Own elaboration from SciMAT data.*

**TABLE 3**

**TABLE 3 ꟾ** Authors with two or more publications.

| **Authors with two or more publications** | | | | | | | |
| --- | --- | --- | --- | --- | --- | --- | --- |
| **Author** | | **Number of articles** | | | **Year of first and last publication** | | **Cited by** |
| Goh, Edmund | | 3 | | | 2018-2020 | | 92 |
| Bejtkovsky, Jiri | | 2 | | | 2013-2016 | | 17 |
| Buenadicha-Mateos, Maria | | 2 | | | 2019 | | 7 |
| Mahmoud, Ali B. | | 2 | | | 2021a,b | | 5 |
| Reisel, William D. | | 2 | | | 2021a,b | | 5 |
| Fuxman, Leonora | | 2 | | | 2021a,b | | 5 |
| Mohr, Iris | | 2 | | | 2021a,b | | 5 |
| **Authors with more than 10 citations** | | | | | | | |
| **Ranking** | **Author** | | **Cited by** | **Year of first and last publication** | | **Number of articles** | |
| 1 | Goh, Edmund | | 92 | 2018-2020 | | 3 | |
| 2 | Lee, Cindy | | 56 | 2018 | | 1 | |
| 3 | Ozkan, Mustafa | | 27 | 2015 | | 1 | |
| 3 | Solmaz, Betul | | 27 | 2015 | | 1 | |
| 4 | Okumus, Fevzi | | 18 | 2020 | | 1 | |
| 4 | Schroth, Holly | | 18 | 2019 | | 1 | |
| 4 | Kong, Sandra | | 18 | 2018 | | 1 | |
| 5 | Bejtkovsky, Jiri | | 17 | 2013-2016 | | 2 | |
| 6 | Lazanyi, K. | | 15 | 2017 | | 1 | |
| 6 | Bilan, Y. | | 15 | 2017 | | 1 | |
| 7 | Christensen, Scott S. | | 12 | 2018 | | 1 | |
| 7 | Wilson, Barbara L. | | 12 | 2018 | | 1 | |
| 7 | Edelman, Linda S. | | 12 | 2018 | | 1 | |

*Source: Own elaboration from SciMAT data.*

**TABLE 4**

**TABLE 4** ꟾ Journals with most impact.

| **Journal** | **Quartile** | **Number of documents** | **Number of citations** | **JIF** |
| --- | --- | --- | --- | --- |
| International Journal of Hospitality Management | Q1 | 2 | 58 | 6.701 |
| California Management Review | Q2 | 1 | 18 | 3.909 |
| Journal of Competitiveness | Q2 | 1 | 17 | 3.649 |
| Tourism and Management Perspectives | Q2 | 1 | 18 | 3.648 |
| International Journal of Environmental Research and Public Health | Q2 | 1 | 7 | 2.849 |
| Sustainability | Q2 | 4 | 14 | 2.576 |
| International Journal of Management Education | Q3 | 1 | 5 | 2.354 |
| Journal of Nursing Management | Q3 | 1 | 12 | 2.243 |
| Frontiers in Psychology | Q2 | 1 | 1 | 2.067 |
| Transformations in Business & Economics | Q4 | 1 | 4 | 1.621 |
| Scandinavian Journal of Psychology | Q2 | 1 | 2 | 1.570 |
| Anales de Psicologia | Q3 | 1 | 1 | 1.346 |
| Journal of Nursing Administration | Q3 | 1 | 4 | 1.274 |
| Journal of Organizational Change Management | Q4 | 1 | 4 | 0.967 |
| International Journal of Manpower | Q4 | 1 | 3 | 0.953 |
| Journal of Business-to-Business Marketing | Q4 | 1 | 9 | 0.543 |

*Source: Own elaboration from SciMAT data and 2019 Journal Citation Reports.*

**TABLE 5**

**TABLE 5** ꟾ Quantitative factors of the themes and their evolution.

|  | **Centrality** | **Density** | **Documents** | **Citations** | **Average citations** | **H-Index** |
| --- | --- | --- | --- | --- | --- | --- |
| **2009-2018** |  |  |  |  |  |  |
| Generation-Z | 137.14 | 50.83 | 9 | 129 | 14.33 | 6 |
| Workforce | 96.9 | 185 | 3 | 74 | 24.67 | 2 |
| Workplace | 75.35 | 100.47 | 4 | 29 | 7.25 | 2 |
| Diversity | 8 | 50 | 1 | 1 | 1 | 1 |
| **2019** |  |  |  |  |  |  |
| Human-Resource-Management | 216.61 | 26.67 | 11 | 45 | 4.09 | 4 |
| Workplace | 157.92 | 83.53 | 9 | 22 | 2.44 | 3 |
| Performance | 167.01 | 104.94 | 6 | 30 | 5 | 2 |
| Culture And Values | 133.21 | 83.58 | 6 | 12 | 2 | 2 |
| Innovation | 22.75 | 25 | 1 | 1 | 1 | 1 |
| **2020-2021** |  |  |  |  |  |  |
| Generation-Z | 213.71 | 82.9 | 22 | 46 | 2.09 | 4 |
| Workforce | 148.77 | 43.75 | 6 | 27 | 4.5 | 3 |
| Impact | 146.71 | 81.21 | 6 | 7 | 1.17 | 1 |
| Performance | 51.47 | 48.61 | 3 | 1 | 0.33 | 1 |
| Research | 14.41 | 25 | 1 | 4 | 4 | 1 |
| Diversity | 7.32 | 66.67 | 1 | 4 | 4 | 1 |
| Response | 11.05 | 50 | 1 | 0 | 0 | 0 |

*Source: Obtained from SciMAT.*

**TABLE 6**

**TABLE 6 ꟾ** “Workforce” cluster network 2009-2018.

| **Number** | **Node A** | **Node B** | **Weight** |
| --- | --- | --- | --- |
| 1 | Behavior | Hospitality | 1 |
| 2 | Behavior | Industry | 1 |
| 3 | Students | Response | 1 |
| 4 | Hospitality | Industry | 1 |
| 5 | Turnover | Older-Workers | 1 |
| 6 | Turnover | Health | 1 |
| 7 | Older-Workers | Health | 1 |
| 8 | Behavior | Workforce | 0.67 |
| 9 | Hospitality | Workforce | 0.67 |
| 10 | Workforce | Industry | 0.67 |
| 11 | Behavior | Students | 0.5 |
| 12 | Behavior | Response | 0.5 |
| 13 | Behavior | Older-Workers | 0.5 |
| 14 | Behavior | Health | 0.5 |
| 15 | Students | Hospitality | 0.5 |
| 16 | Students | Industry | 0.5 |
| 17 | Hospitality | Turnover | 0.5 |
| 18 | Hospitality | Response | 0.5 |
| 19 | Hospitality | Older-Workers | 0.5 |
| 20 | Hospitality | Health | 0.5 |
| 21 | Turnover | Industry | 0.5 |
| 22 | Industry | Response | 0.5 |
| 23 | Industry | Older-Workers | 0.5 |
| 24 | Industry | Health | 0.5 |

*Source: Elaborated from SciMAT data.*

**TABLE 7**

**Table 7 ꟾ** “Performance” Cluster Network 2019.

| **Number** | **Node A** | **Node B** | **Weight** |
| --- | --- | --- | --- |
| 1 | Entrepreneurship | Turnover | 1 |
| 2 | Outcomes | Psychology | 1 |
| 3 | Commitment | Diversity | 0.5 |
| 4 | Commitment | Outcomes | 0.5 |
| 5 | Commitment | Psychology | 0.5 |
| 6 | Performance | Diversity | 0.5 |
| 7 | Performance | Outcomes | 0.5 |
| 8 | Performance | Psychology | 0.5 |
| 9 | Organisation | Management | 0.4 |

*Source: Elaborated from SciMAT data.*

**TABLE 8**

**TABLE 8 ꟾ** “Generation-Z” cluster network 2020-2021.

| **Number** | **Node A** | **Node B** | **Weight** |
| --- | --- | --- | --- |
| 1 | Generation-Z | Human-Resource-Management | 0.45 |
| 2 | Hospitality | Perceptions | 0.56 |
| 3 | Motivation | Work | 0.46 |

*Source: Elaborated from SciMAT data.*
